# Are Aquaporins Expressed in Stomatal Complexes Promising Targets to Enhance Stomatal Dynamics?

**DOI:** 10.3389/fpls.2020.00458

**Published:** 2020-04-21

**Authors:** Lei Ding, François Chaumont

**Affiliations:** Louvain Institute of Biomolecular Science and Technology, UCLouvain, Louvain-la-Neuve, Belgium

**Keywords:** aquaporin, stomatal movement, signaling, guard cell, membrane diffusion, water, hydrogen peroxide, carbon dioxide

## Abstract

The opening and closure of stomata depend on the turgor pressure adjustment by the influx or efflux of ions and water in guard cells. In this process, aquaporins may play important roles by facilitating the transport of water and other small molecules. In this perspective, we consider the potential roles of aquaporins in the membrane diffusion of different molecules (H_2_O, CO_2_, and H_2_O_2_), processes dependent on abscisic acid and CO_2_ signaling in guard cells. While the limited data already available emphasizes the roles of aquaporins in stomatal movement, we propose additional approaches to elucidate the specific roles of single or several aquaporin isoforms in the stomata and evaluate the perspectives aquaporins might offer to improve stomatal dynamics.

## Introduction

Stomata are pores formed by two guard cells allowing CO_2_ uptake for photosynthesis at the expense of water loss by transpiration. While most stomata are formed by two kidney-shaped guard cells, the stomatal complexes of grass consist of two dumbbell-shaped guard cells flanked by two subsidiary cells. The opening and closure of stomata depend on variations in the turgor pressure of the guard cells and, thus, on water and solute fluxes across the cell plasma membrane. Therefore, due to their channel activity and specificity, aquaporins are thought to be involved in stomatal movement. Plant aquaporins cluster in different subfamilies, including the plasma membrane intrinsic proteins (PIPs) and the tonoplast intrinsic proteins (TIPs). In addition to their water channel activity, aquaporins can also facilitate the diffusion of various other small solutes, including H_2_O_2_ and CO_2_, two important signaling molecules in guard cells. Several aquaporins belonging to the PIP and TIP subfamilies have been reported to be expressed in guard cells or stomatal complexes in various plant species (Chen et al., 2017; Hachez et al., 2017). For instance, *PIP* aquaporin gene expression was analyzed in maize stomatal complexes isolated by laser microdissection (Heinen et al., 2014). The expression of seven *PIP* genes accounts for more than 98% of the total *PIP* transcripts and the expression of most of them follows a diurnal pattern (Heinen et al., 2014). Diurnal variation in *PIP* and *TIP* transcript abundance is also detected in the guard cells of *Populus* tree using a similar microdissection technique (Durand et al., 2020).

Several papers investigating the physiological role and regulation of the aquaporins expressed in the stomatal complexes have been recently published and the data reviewed by us and colleagues (reviewed in Maurel et al., 2016; Chen et al., 2017; Hachez et al., 2017; Nunes et al., 2019). The aim of this perspective paper is to focus on putative specific roles of aquaporins in stomatal complexes, present the future challenge to elucidate them, and evaluate the pertinence to target aquaporins to improve stomatal function.

## Assessing the Direct or Indirect Role of Aquaporins in Stomatal Conductance

**FIGURE 1:**
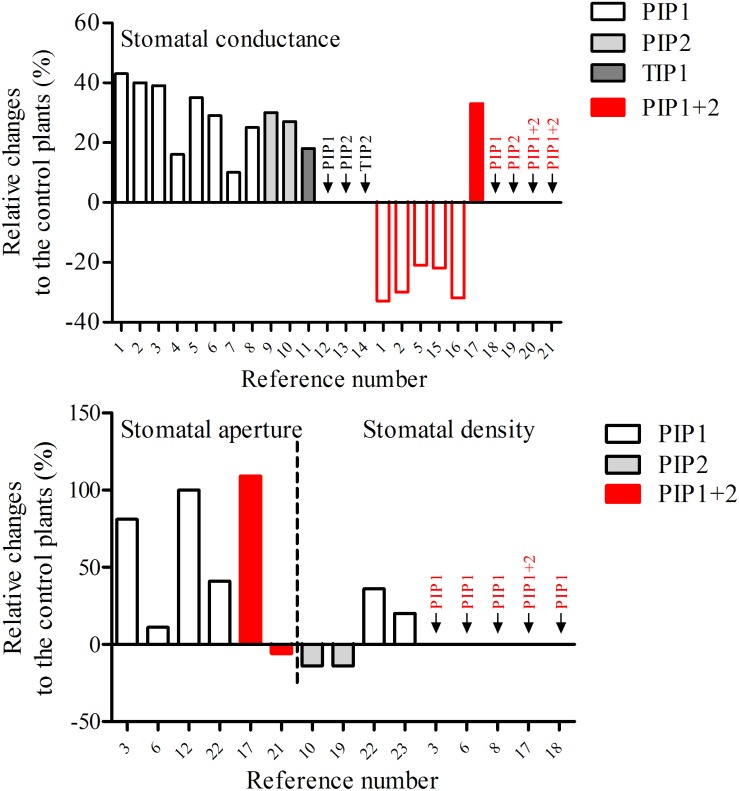
Deregulation of aquaporin expression and its effect on stomatal conductance, stomatal aperture, and stomatal density. Black bar borders indicate overexpression of aquaporins. Red bar borders indicate silencing of aquaporins. The numbers refer to the references. All the overexpression results were obtained with the use of constitutive promoters. References: 1, *Nicotiana tabacum*, Uehlein et al. (2003); 2, *Nicotiana tabacum*, Flexas et al. (2006); 3, *Nicotiana tabacum*, Sade et al. (2010); 4, *Nicotiana tabacum*, Kawase et al. (2013); 5, *Arabidopsis thaliana*, Sade et al. (2014); 6, *Glycine max*, Zhou et al. (2014); 7, *Oryza sativa*, Li et al. (2018); 8, *Oryza sativa*, Xu et al. (2019); 9, *Oryza sativa*, Nada and Abogadallah (2020); 10, *Oryza sativa*, Hanba et al. (2004); 11, *Arabidopsis thaliana*, Lin et al. (2007); 12, *Solanum lycopersicum*, Wang et al. (2017); 13, *Populus tremula* × *Populus alba*, Ranganathan et al. (2017); 14, *Nicotiana tabacum*, Martins et al. (2017); 15, *Nicotiana tabacum*, Uehlein et al. (2008); 16, *Oryza sativa*, Ding et al. (2016); 17, *Populus tremula* × *Populus alba*, Bi et al. (2015); 18, *Arabidopsis thaliana*, Heckwolf et al. (2011); 19, *Arabidopsis thaliana*, Li et al. (2015); 20, *Arabidopsis thaliana*, Martre et al. (2002); 21, *Arabidopsis thaliana*, Ceciliato et al. (2019); 22, *Nicotiana tabacum*, Ding et al. (2004); 23, *Nicotiana tabacum*, Aharon et al. (2003).

The role of specific aquaporins in stomatal complexes can be assessed from reverse genetic approaches, either by overexpressing or silencing/knocking out a single gene. In general, deregulating aquaporin expression affects the photosynthesis, the stomatal conductance (*g*_s_), the stomatal movement and/or density, as well as the plant hydraulics (for review, see Maurel et al., 2016; Hachez et al., 2017). As illustrated in Figure 1 and Table 1, most of the studies showed an increase in *g*_s_ for both monocot and dicot plants overexpressing aquaporin genes, including *PIP1*, *PIP2*, and *TIP*, under control growth condition, while a decrease in *g*_s_ was recorded in *PIP* silenced plants. However, it is important to emphasize that this deregulation of aquaporin gene expression is generally not restricted to the guard cells: most aquaporins are expressed widely throughout the plant and their silencing can affect different tissues. Similarly, the promoters used to overexpress *PIP* genes are very often constitutive such as the cauliflower mosaic virus *35S* promoter or the ubiquitin promoter. The *g*_s_ is regulated by different mechanisms and signaling events at the whole plant level involving root to shoot communication through hydraulic and chemical signals (Comstock, 2002; Parent et al., 2009; Tardieu et al., 2015) or the photosynthetic apparatus (Lawson et al., 2014; Sade et al., 2014). It is therefore very difficult to determine whether the observed phenotypes are related to altered aquaporin activity within the guard cells themselves or in other root or leaf tissues. This is illustrated by the study of Sade et al. (2014), who showed that the *g*_s_ as well as the photosynthetic rate and mesophyll conductance increase in *Arabidopsis* lines overexpressing *Nt*AQP1 (a PIP isoform) under the control of the *35S* promoter or the mainly photosynthetic tissue promoter *FBPase*, but not in lines in which *Nt*AQP1 is expressed under the control of the stomatal-specific promoter *KST1* (Sade et al., 2014). This data indicates that aquaporins can indirectly affect the *g*_s_ through changes in tissue hydraulic properties probably affecting signaling processes.

**TABLE 1:** The relationship between aquaporins and stomatal movement.

**Genes**	**Localization**	**Species**	**Regulation**	**Substrate**	**References**
Sun*TIP7*	Guard cell	Sunflower	Diurnal rhythm of expression		Sarda et al. (1997)
*VfPIP1* OE	Epidermal peels	*Arabidopsis*	Stomata closed faster under ABA and dark treatments		Cui et al. (2008)
*Atpip2;1* KO	Epidermal peels		Stomata closed slower under ABA treatment, no difference under [CO_2_] changing	H_2_O and H_2_O_2_	Grondin et al. (2015)
*Atpip2;1* KO	Epidermal peels + intact leaf		Stomatal aperture and movement no differences under [CO_2_] changing and ABA treatments	H_2_O and CO_2_	Wang et al. (2016)
*Atpip2;1* KO	Epidermal peels		Stomata closed slower under ABA and flg22 treatments	H_2_O and H_2_O_2_	Rodrigues et al. (2017)
*Atpip1;1/pip1;2/pip2;1/pip2;2* KO	Epidermal peels + intact leaf		Stomatal aperture and movement no differences under ABA treatment		Ceciliato et al. (2019)
*ZmPIP*s	Stomata	Maize	Diurnal rhythm of expression	H_2_O and CO_2_	Heinen et al. (2014)
*ZmPIP*s	Intact leaf		Expression regulated by [CO_2_] changing and carbonic anhydrase activity		Kolbe et al. (2019)
*MdPIP1;3* OE	Intact leaf	Tomato	Stomata closed faster under drought stress		Wang et al. (2017)
*GmPIP1;6* OE	Intact leaf	Soybean	Stomata closed slower under salt stress		Zhou et al. (2014)
*PIPs & TIPs*	Stomata	*Populus*	Expression regulated by diurnal rhythm and water stress		Durand et al. (2020)

*The PIP and TIP isoforms are constitutively expressed under the control of the 35S promoter and silenced by transposon or T-DNA insertion.*

In addition, the *g*_s_ is determined by the stomatal anatomy, including the stomatal density, the size and the pore area (Franks and Beerling, 2009; Faralli et al., 2019), which are potentially influenced by aquaporin expression (Figure 1). The high *g*_s_ in aquaporin overexpressing lines may be associated with the increase in the stomatal density and/or the stomatal aperture. However, the development of stomata is controlled by complex cellular processes (Chater et al., 2017), and it remains unclear if/how stomatal development/density depends on aquaporins. As shown in Figure 1, the stomatal aperture is increased by overexpressing PIP1 isoforms, while the stomatal density is less affected by the deregulation of PIP1s and/or PIP2s.

## Roles of Aquaporins Expressed in the Stomatal Complexes

Ideally, the role of aquaporins expressed in the stomatal complexes has to be determined from plant lines in which aquaporin expression is specifically modified in the guard cells and/or the subsidiary cells. This can be achieved by using cell specific promoters. Similarly, aquaporin silencing through cell type-specific CRISPR-TSKO technology can now be applied (Decaestecker et al., 2019). As mentioned above, Sade et al. (2014) expressed *Nt*AQP1 under the control of the guard cell specific promoter *KST1* in *Arabidopsis*, but did not observe any change in *g*_s_, even if they did not investigate other stomatal behaviors, including the kinetic of stomatal opening and closure. However, plant lines constitutively deregulated in aquaporin gene expression already available can also constitute a good starting material to obtain epidermal peels and follow the stomatal behavior in different conditions. This approach has been recently used to decipher the role and regulation of *Arabidopsis* PIP aquaporins in guard cells, in relation to the transport of water, CO_2_ and H_2_O_2_.

### Water

To control the opening and closure of stomata, guard cells have to adjust their volume by up to ∼50% driven by the accumulation and release of solutes, respectively, as well as by the bidirectional transport of water through the plasma membrane and the tonoplast (Jezek and Blatt, 2017). Interestingly, in the grass species having two closely associated lateral subsidiary cells, an inverse behavior of the solute exchanges through the membranes of the subsidiary cells compared with the guard cells controls the opening and closure of the stomata (Chen et al., 2017; Nunes et al., 2019).

Evidence for the involvement of PIP aquaporins in water exchange came from Grondin et al. (2015) and Rodrigues et al. (2017). They showed that the water permeability of wild type guard cell protoplasts is significantly enhanced by abscisic acid (ABA) and the pathogen-associated molecular pattern peptide flg22, which is a well-known molecule inducing stomatal closure, whereas this increase was not observed in guard cells from *pip2;1* KO plants. The closure of stomata observed on peeled epidermis was also reduced in *pip2;1* KO lines upon ABA or flg22 incubation (Grondin et al., 2015; Rodrigues et al., 2017). Furthermore ABA treatment activated OST1 kinase, and OST1 phosphorylates Ser-121 of PIP2;1, an event known to activate the water channel activity of PIP aquaporins (Grondin et al., 2015). Altogether, these results suggest that ABA-induced stomatal closure in *Arabidopsis* is hydraulically controlled by aquaporins, including PIP2;1 and possibly other PIPs and that PIPs are involved in the stomatal kinetics. However, this data could not be reproduced in an independent study: stomata from *pip2;1* KO line retained wild-type like ABA-induced stomatal closure response (Wang et al., 2016). The authors speculated that overlapping aquaporin functions may exist in guard cells since different *PIP* genes are expressed in *Arabidopsis* stomata (Zhao et al., 2008). Later on, a quadruple *pip1;1*, *pip1;2*, *pip2;1*, and *pip2;2* mutant was generated by the same group, but no significant difference in the decrease in stomatal aperture and *g*_s_ upon ABA treatment was observed between the WT and the quadruple mutant (Ceciliato et al., 2019).

Such contradictory results could be explained by different experimental growth conditions or measurement methods. Additional experiments in *Arabidopsis* or other species are definitely required to confirm or infirm the involvement of specific PIP aquaporins in the dynamic of stomatal movement. This is currently investigated in our laboratory in maize lines deregulated in PIP expression (Ding et al., 2020 and unpublished data).

### CO_2_

It is well known that high CO_2_ concentration ([CO_2_]) and low [CO_2_] induce stomatal closure and opening, respectively (Zhang et al., 2018). As mentioned above, several PIP aquaporins have been shown to facilitate membrane CO_2_ diffusion. It is the case for the *Arabidopsis* PIP2;1 when expressed in *Xenopus* oocytes, suggesting that PIP2;1 facilitates the entry of CO_2_ into the guard cells to trigger downstream signaling leading to stomatal closure (Wang et al., 2016). PIP2;1 also interacts with the carbonic anhydrase (CA) βCA4, and this interaction enables the extracellular CO_2_ enhancement of the S-type anion channel SLAC1 activity in oocytes, due to the influx of CO_2_ through PIP2;1 and the production of intracellular HCO_3_^–^, known as a second messenger activating the SLAC1 activity (Xue et al., 2011; Wang et al., 2016; Zhang et al., 2018). However, no change in CO_2_ regulation of stomatal movement was observed in *pip2;1* mutant compared to the WT, suggesting again that other PIP aquaporins expressed in *Arabidopsis* guard cells could have similar roles (Wang et al., 2016). In maize, several *PIP* genes are expressed in stomatal complexes and both PIP1;5 and PIP1;6 are able to increase the membrane CO_2_ permeability when expressed in yeast (Heinen et al., 2014). The difficulty of gene redundancy could be overcome by specifically targeting the silencing of several *PIP* genes in guard cells with the CRISPR-TSKO technique mentioned above. The expression of *PIP* genes was analyzed in maize *ca* mutants growing under high and low [CO_2_] (Kolbe et al., 2019). Interestingly, PIP1;2 and PIP2;5 were up- and down-regulated, respectively, in both WT and *ca1ca2* mutant by a low [CO_2_] treatment. The observation that PIP1;2 expression increases in *ca* mutants or under low [CO_2_] suggests that PIP1;2 may also act as CO_2_ membrane facilitator in maize leaves. The fact that these *PIP1;2* and *PIP2;5* genes do not have a consistent response to CO_2_ is probably due to different physiological roles, i.e., in controlling CO_2_ and/or water movement at low [CO_2_]. However, this study investigates the expression of PIPs in whole leaves, and it is difficult to distinguish their respective roles in stomatal complexes or other leaf tissues.

### H_2_O_2_

Reactive oxygen species (ROS) play important roles in stomatal closure induced by high [CO_2_], ABA and biotic stress (Chater et al., 2015; Rodrigues et al., 2017; Sussmilch et al., 2019). H_2_O_2_ is produced in the apoplasm by the activated NADPH oxidases and acts in the guard cells to regulate the activity of Ca^2+^ channels leading to the activation of SLAC1 in the plasma membrane (Pei et al., 2000). Several aquaporins were previously characterized as H_2_O_2_ channels when expressed in yeast (Bienert et al., 2007, 2014; Bienert and Chaumont, 2014) or reconstituted into liposomes (Wang et al., 2020), or in planta in association with abiotic (Jang et al., 2012; Smirnoff and Arnaud, 2019) and biotic stress (Tian et al., 2016). In *Arabidopsis pip2;1* mutant, a lack of ROS (H_2_O_2_) accumulation in guard cells, was observed upon ABA and flg22 treatments (Grondin et al., 2015; Rodrigues et al., 2017), suggesting that PIP aquaporins facilitate the membrane diffusion of H_2_O_2_ to regulate the stomatal movement.

## Do Aquaporins Directly or Indirectly Affect Cation Transport in Stomatal Complexes?

Changes in cell volume depend on the cell osmolality and the addition or the removal of membrane materials. This process in guard cells integrates the membrane traffic with ion transport [reviewed in Jezek and Blatt (2017)]. The soluble N-*ethylmaleimide*-sensitive factor attachment protein receptor (SNARE) syntaxin SYP121 was shown to regulate the activity of K^+^ channels (Eisenach et al., 2012). In *Arabidopsis syp121* mutant, the stomatal reopening following closure in elevated [Ca^2+^] is slower than in WT plants, due to a reduction of the recycling of KAT1 from endosomal membranes. In addition, SYP121 physically interacts with the voltage sensor domain of the K^+^ channels KC1 and KAT1, promoting the channel activity. Hence, SYP121 appears as a major regulator of the membrane voltage sensing to coordinate the rate of the secretory traffic with the K^+^ transport (Grefen et al., 2015; Jezek and Blatt, 2017). Interestingly, SYP121 also physically interacts with PIP aquaporins in maize and *Arabidopsis* (Besserer et al., 2012; Hachez et al., 2014). This interaction is required for the delivery of PIPs to the plasma membrane and might also affect their water channel activity. Mesophyll protoplasts have indeed a lower membrane water permeability in *syp121* mutant than in WT plants (Besserer et al., 2012), indicating that SYP121 is necessary for PIPs trafficking to the plasma membrane. It is therefore tempting to speculate that SYP121 controls the trafficking and activity of both K^+^ and water channels in a coordinated way to regulate the cell volume during the stomatal movement. In addition, in other parts of the plants, expression data indicates that K^+^ concentration directly affects the expression of PIP aquaporins, K^+^ starvation reducing their expression while K^+^ resupply increasing the expression (Maathuis et al., 2003; Armengaud et al., 2004). K^+^ channel inhibitors also decreased both water flow and expression of PIP aquaporins (Tazawa et al., 2001; Sahr et al., 2005). Finally, there is still an ancient but still valid hypothesis that PIP aquaporins could serve as turgor sensors in the plasma membrane to modulate the K^+^ channel activity and cell water homeostasis (Hill et al., 2004; Nongpiur et al., 2020).

Regulation of PIP trafficking appears even more complex. PIPs in higher plants are phylogenetically divided into two groups, PIP1 and PIP2, showing different subcellular localization and activities in protoplasts or when expressed in heterologous systems. While most PIP1s are retained in the endoplasmic reticulum (ER), PIP2s are found in the plasma membrane, but PIP1s and PIP2s can assemble as heterotetramers resulting in co-localization of both proteins in the plasma membrane and an increase in the cell membrane water permeability (Fetter et al., 2004; Berny et al., 2016; Jozefkowicz et al., 2017). In maize stomatal complexes, the proportion of *PIP1* transcripts accounts for up to 85% of the total abundant *PIP* transcripts, raising the question whether all PIP1s are located in the cell plasma membrane (Heinen et al., 2014). By definition, as PIPs assemble in heterotetramers in all the stoichiometries (Berny et al., 2016), one PIP2 isoform would be enough to direct three PIP1s to the plasma membrane. In this context, the regulation of the subcellular trafficking of PIPs in guard cells and subsidiary cells according to the environmental conditions (day/night, ABA, CO_2_) has to be investigated.

Finally, it was recently reported that *Arabidopsis* PIP2;1 and PIP2;2 can function as Na^+^ channels in addition to water channels (Byrt et al., 2017; Kourghi et al., 2017). This finding challenges the concept of plant aquaporins as channels of small uncharged solutes. Even though Na^+^ is not the main element in guard cells, it could participate in stomatal control, as suggested in poplars in which the higher Na^+^ amount in guard cells of hybrid poplars correlated with higher levels of the vacuolar Na^+^/proton antiporter NHX1.13 and some PIP and TIP aquaporins (Durand et al., 2020). Whether PIP or TIP aquaporins expressed in stomatal complexes increase permeability to Na^+^ and directly contribute to the stomatal movement remains to be elucidated.

## Conclusion

The expression of several *PIP* and/or *TIP* aquaporin genes in stomatal complexes is now well documented (Chen et al., 2017). The constitutive deregulation of aquaporin expression in the whole plant definitely demonstrates the involvement of aquaporins in stomatal conductance, transpiration and photosynthesis, but very limited studies focus on the physiological roles of specific isoform in the guard cells and/or subsidiary cells. Results obtained in *Arabidopsis* indicate that PIP2;1 aquaporin participates in ABA signaling in guard cells leading to stomatal closure by facilitating H_2_O_2_ entrance and water exit (Grondin et al., 2015; Rodrigues et al., 2017). PIP2;1 is also suggested to be involved in CO_2_ signaling through its transport and direct interaction with carbonic anhydrase (Wang et al., 2016). These observations indicate that aquaporins could be an important link between the ABA or CO_2_ signaling pathway and the cell ROS distribution inducing the stomatal closure. It is interesting to observe that aquaporins act in the kinetics of stomatal closure in *Arabidopsis* (Grondin et al., 2015) or in maize (Ding and Chaumont, unpublished data). It was recently shown that expression of light-gated K^+^ channel BLINK1 in guard cells accelerates the stomatal aperture and closure, improving carbon assimilation, water use and plant growth (Papanatsiou et al., 2019). Therefore, optimizing the expression and/or activity of aquaporins that affect the kinetics of stomatal closure may represent another interesting way to reach similar favorable phenotypes in crops facing ever-changing environmental conditions. To reach this goal, a better understanding of the contribution and regulation (activity, trafficking) of each aquaporin isoform expressed in stomatal complexes is still required, including the TIPs that are located in the tonoplast. This systematic characterization is important to identify the most promising aquaporin isoforms and understand their contribution to the stomatal dynamic behavior in relation to the signaling molecules and, in turn, to improve the water use efficiency.

## Data Availability Statement

The datasets generated for this study are available on request to the corresponding author.

## Author Contributions

Both authors contributed to the writing of the manuscript.

## Conflict of Interest

The authors declare that the research was conducted in the absence of any commercial or financial relationships that could be construed as a potential conflict of interest.
